# Structural Equation Modeling on Health-related Quality of Life of Patients with Ankylosing Spondylitis

**Published:** 2017-10

**Authors:** Ae Ri JANG, Keum Seong JANG

**Affiliations:** 1. Dept. of Nursing, Songwon University, Gwangju, Korea; 2. College of Nursing, Chonnam National University, Gwangju, Korea

**Keywords:** Ankylosing spondylitis, Environmental support, Depression, Function

## Abstract

**Background:**

Ankylosing Spondylitis (AS) is a chronic, progressive, and inflammatory disease. The objective of this study was to construct a hypothetical model to determine factors affecting health-related quality of life (HRQOL) of AS patients (ASHRQOL).

**Methods:**

A survey was conducted on 275 patients who visited Chonnam National University Bitgoeul Hospital. Data of different variables were collected over two months in 2015.

**Results:**

A hypothetical model did not reach recommended level of fit verification. Therefore, health perception variable was eliminated on theoretical basis. Path between function and HRQOL was added after eliminating unimportant path before completing the final modified model.

**Conclusion:**

The final revised model met recommended levels of fit test except AGFI. The model explained 57.6% of ASQOL. Further study is needed to develop efficient interventional strategy for improving ASHRQOL.

## Introduction

Ankylosing Spondylitis (AS) is a chronic, progressive, and inflammatory disease that can cause stiffness and pain on the back and peripheral joints. It can also cause inflammation of body parts (e.g., eyes, heart, lungs) and changes to body structure involving the axial skeleton (1). Morbidity of AS has been reported to be approximately 0.1–0.4% in the US (2). However, its actual rate could be higher since AS is easily misinterpreted as other diseases or simple back pain (3).

Factors affecting QOL include personal factors (4–8) such as smoking and sex, symptomatic factors (3,9,10) such as pain, stiffness, fatigue, quality of sleep (QOS), depression, and function (6,11,12), bio-physiological factors (13,14) such as hematological findings and BMI, nonpharmacological factors (15) such as exercise and hot spring therapy, cognitive factors (16) including self-awareness, and environmental factors (17) including social support.

However, previous studies do not reflect symptomatic effects on AS patients’ daily performance since they measure other parameters of QOL without theoretical basis. Instead, they predict HRQOL based on simple inter-factorial equations for each independent factor. Therefore, various factors need to be explored in order to improve AS patients’ HRQOL based on relevant HRQOL model and validate each factor in a multi-dimensional and integral way.

All factors are verified mainly in chronic patients using Wilson & Cleary’s HRQOL model (18–20). This model is very useful since it reflects general characteristics of AS patients. This model also includes personal and environmental factors that reflect disease characteristics, symptoms, function, health awareness, and concept of HRQOL. This study was based on that structuring model to explain HRQOL of AS patients (ASHRQOL). Personal factors such as sex, smoking status, economic status, education, occupation, duration of illness, and duration of symptoms must be considered as factors of ASHRQOL (4–8,21–23). However, using categorically independent variable as a dummy variable is not recommended or practical (24). Thus, variables used in the structural model were limited to age, economic status, and smoking, the most powerful variables related to disease specificity. Smoking frequency (smoking) rather than smoking duration was validated as a key factor in this study because smoking intensity was more harmful than smoking duration (5).

C-reactive protein (CRP), erythrocyte sedimentation rate (ESR), and body mass index (BMI) are factors that affect QOL (14). In this study, CRP and ESR were validated as bio-physiological factors. Fatigue exerts negative effects on the quality of life of patients with ankylosing spondylitis (25). It also increases the limitation of their function (10). Nocturnal and general pain is strongly correlated with function, disease activity, and ASHRQOL (26). Morning stiffness also shows a significant correlation with HRQOL and the number of edematous joints of AS patients (27).

Anxiety and depression can significantly worsen disease activity and functional limitations (9,28). In this study, fatigue, pain, and morning stiffness were validated as symptomatic factors affecting QOL. Depression was parametrized based on results of many studies showing that depression and anxiety had direct impact on quality of life. Anxiety items were included in depression assessment tools (29).

Physical activity has shown positive effects on symptoms, functioning, and ASHRQOL (30–32). According to a meta-analysis of 10 papers, physical therapy can affect AS patients (33). Therefore, non-pharmaceutical factors were measured by physical activity.

The aim of this study was to construct a hypothetical model to determine factors affecting health-related quality of life (HRQOL) of AS patients (ASHRQOL).

## Materials and Methods

### Samples

This study used a cross-sectional design. A minimum of > 250 cases were needed in this study considering the characteristic of AS being a rare incurable disease (24).

A total of 75 cases were used in our final analysis (282 questionnaires were distributed). All participants > 19 years old were diagnosed as AS. They were on drug therapy for at least four weeks. Patients provided informed consent before participating in this study. Of these, five cases dropped out and two cases were not analyzed because patients had > 10% missing values for each measured variable due to their insincere replies. These seven cases were excluded from analysis.

### Data Collection

All procedures performed in this study involving human participants were in accordance with ethical standards. All data were collected after obtaining approval from Chonnam National University Hospital Biomedical Research Ethics Committee (Approval number CNUH-2015-009).

This study was conducted at a rheumatology out-patient clinic in Chonnam University Hospital. Data were collected during a 2-month period in 2015. All researchers and trained data handlers were posted at the out-patient clinic after receiving approval before conducting this study.

In-body measurements for height, weight, and blood test for CRP and ESR were performed within two months after commencing this study. In each questionnaire, participants were required to answer all questions individually after reading each question (or after question were read to them and written by data handlers on request). Researchers recorded smoking, alcohol drinking, period after diagnosis, and medication through direct interviews or e-chats. If there were insufficient or omitted details, information was supplemented by direct contact between data handler and patient.

### Data analysis

IBM SPSS Statistics 20.0® (Chicago, IL, USA) and IBM SPSS AMOS 21.0® were used for data analysis. General characteristics and clinical characteristics of subjects were analyzed by descriptive statistics. Normality of descriptive statistics for observed variables was verified using median, mean, skewness, and kurtosis. Pearson’s correlation coefficient was used to analyze relations between major key factors. Confirmatory factor analysis was used to identify the validity of latent variables. Structural equation modeling was used to verify the QOL model of ankylosing spondylitis patients and indirect/direct path coefficients of factors affecting ASHRQOL. Maximum likelihood that postulated multivariate normality was used to verify model structuring. To verify the fitness of the proposed model, χ^2^ values, absolute fit index (GFI), modified fit index (AGFI), elemental mean square approximation error (RMSEA), suitable index (CFI), standard fit index (NFI), and Tucker-Lewis index suitable (TLI) were used.

### Validity, reliability, and rigor

In this study, Cronbach’s α value on environmental support was 0.972 using Medical Outcome Study Social Support Survey (34). Cronbach’s α on symptoms was 0.92 using the Korean version Bath Ankylosing Spondylitis Disease Activity Index (35). Cronbach’s α value on depression was 0.923 using Korean Beck Depression Index-II (29). Cronbach’s α on function was 0.961 using Korean Bath Ankylosing Spondylitis Functional Index. Measure of health awareness was scored according to Status of Health using JH Oh’s 1-point question tool adapted from the “Health Perception Questionnaire” sub-standard. Measure of physical activity was converted as successive score of MET (min/week) using Korean International Physical Activity Index. Cronbach’s α value on HRQOL was 0.799 using Korean ASAS-HI (36).

## Results

The mean age of study subjects was 40 years (range, 19 to 77 years). The skewness was below ±2.0 and kurtosis was below 10.0 for all measured factors except CRP and physical activity, which satisfied the hypothesis of normal distribution. Skewness and kurtosis for CRP were 5.950 and 45.68, respectively. They were 3.67 and 17.71 for physical activity, respectively, which did not satisfy any hypothetical normal distribution. Thus, log-transformed statistical analysis was performed (Table 1).

**Table 1:** Descriptive statistics of the measured variables (n=275)

***Variables***	***Mean±SD (Range)***	***Skewness***	***Kurtosis***
Smoking intensity	0.23±0.41(0–2)	1.612	1.889
CRP^a^	0.56±1.20(0–12)	5.95	45.68
ESR^b^	15.53±14.82(0–80)	1.70	3.121
Material support	78.65±17.35(20–100)	−0.90	0.71
Affectionate support	78.90±17.88(20–100)	−0.94	0.80
Positive Social support	79.63±18.18(20–100)	−0.86	0.40
Information Emotional support	75.64±18.49(20–100)	−0.61	−0.03
Physical Activity	5172.56±4405.91(0–32880)	3.67	17.71
Fatigue	4.65±2.48(0–10)	−0.61	−0.84
Pain	9.73±7.21(0–30)	0.59	−0.42
Stiffness	6.73±5.38(0–20)	0.653	−0.57
Depression	13.59±9.60(0–58)	1.14	2.13
Function	17.10±22.72(0–96.60)	1.72	2.27
Health perception	2.67±0.14(1–5)	0.14	−0.02
HRQOL^c^	5.70±4.04(0–17)	0.58	−0.30

a CRP=C-reactive protein,

b ESR=Erythrocyte sedimentation rate,

c HRQOL=Health-related Quality of Life

Factors that showed inter-factorial correlation coefficient > 0.75 included material and affectionate support, positive social support, informational and emotional support, affectionate and positive social support, informational and emotional support, positive social and informational emotional support, and pain and stiffness. Affectionate and positive social support along with informational and emotional support was possibly related to latent variable of environmental support. Pain and stiffness were tied to latent variable of symptom. Results from this study indicated that there was no multicollinearity between independent factors.

**Fig. 1:**
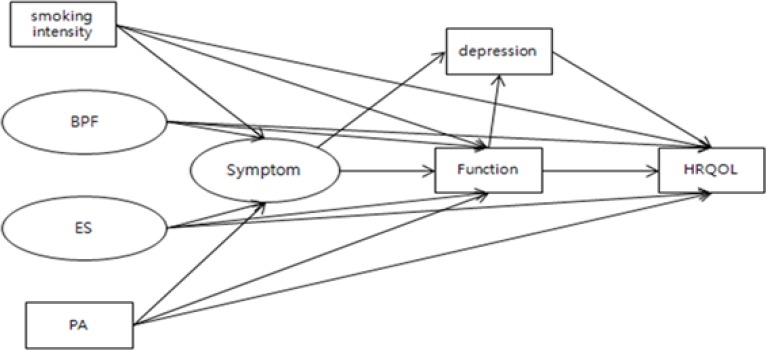
Hypothetical model BPF=Biological Physiological Factor, ES=Environmental Support, PA=Physical Activity, HRQOL=Health related Quality of Life

Test results of the original hypothetical model (Fig. 1) were χ^2^ = 260.602 (p < 0.001, df = 70), GFI = 0.895, AGFI = 0.821, RMSEA = 0.278, CFI = 0.920, NFI = 0.895. Results did not necessarily meet the recommended level of confidence of fitness index (FI). Thus, the original model was modified to achieve a simpler model with a sufficiently high FI. The index correction model met all satisfactory levels of FI confidence with the following results: χ^2^ = 164.634 (p < 0.001, df = 57), GFI = 0.916, AGFI = 0.866, RMSEA = 0.083, CFI = 0.953, NFI = 0.930, and TLI = 0.936.

**Fig. 2:**
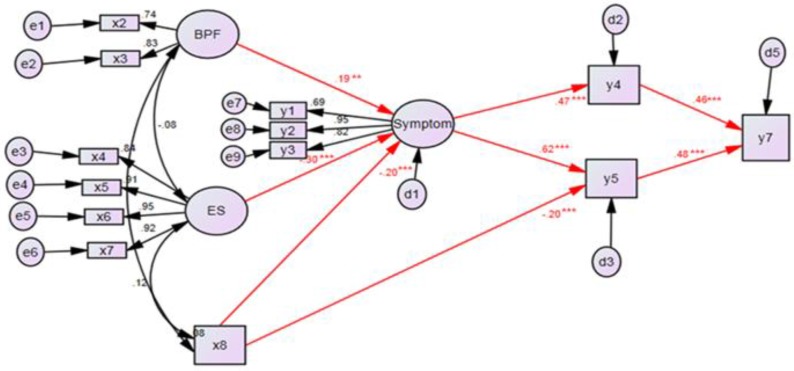
Hypothetical model x2=C-reactive protein, x3=Erythrocyte sedimentation rate, x4=Material support, x5=Affectionate support, x6=Positive Social support, x7=Information Emotional support, x8=Physical Activity, y1=Fatigue, y2=Pain, y3=Stiffness, y4=Depression, y5=Function, y7=Health related quality of life, BPF=Biological Physical Factor, ES=Environmental Support, e1∼e9=Measurement error, d1,2,3,5=Structural error. ***: *P*<0.001, **: *P*<0.01, *: *P*<0.05

Bootstrapping® program (IBM SPSS AMOS 21.0) was used for indirect-effect, direct-effect, and total-effect analysis of the final correction model. Results indicated that, among factors affecting AS HRQOL, physiological factors had direct effect of 0.192 (*P* < 0.01), environmental support had negative direct effect of 0.303 (*P* < 0.01), physical activity had negative direct effect of 0.197 (*P* < 0.01) on symptoms, while symptoms had direct effect of 0.474 (*P* < 0.01) on depression. Symptoms also showed direct effect of 0.625 on function. Physical activity showed negative direct effect of 0.204, negative indirect effect of 0.123, and negative total effect of 0.327 on function. Depression showed direct effect of 0.459 while function showed direct effect of 0.475 on AS HRQOL with statistically satisfactory level of confidence. Among 20 hypotheses and hypothetical models, 13 were eliminated and one was newly established. Eight hypotheses were verified to have statistically satisfactory levels of confidence (Fig. 2). Detailed results are shown in Table 2.

**Table 2:** Hypothesis of the final modified model

***Endogenous variable***	***Hypothesis***		***Final Modified Model result***	
Symptom	H1	Smoking intensity could effect on symptom. (remove)		
	H2	Physiological factor could effect on symptom.	H1	support
	H3	Environmental support could effect on symptom.	H2	support
	H4	Physical activity could effect on symptom.	H3	support
Depression	H5	Symptom could effect on depression.	H4	support
	H6	Depression could effect on function. (remove)		
	H7	Smoking intensity could effect on function. (remove)		
	H8	Physiological facto could effect on function r. (remove)		
Function	H9	Symptom could effect on function.	H5	support
	H10	Environmental support could effect on function.		
	H11	Physical activity could effect on function.	H6	support
	H12	Depression could effect on health awareness. (remove)		
Health perception	H13	Smoking intensity could effect on health awareness. (remove)		
	H14	Function could effect on health awareness. (remove)		
	H15	Environmental support could effect on health awareness. (remove)		
Health-related Quality of Life	H16	Depression could effect on HRQOL.	H7	support
	H17	Smoking intensity could effect on HRQOL. (remove)		
	H18	Health awareness could effect on HRQOL. (remove)		
	H19	Environmental support could effect on HRQOL. (remove)		

## Discussion

The final model proposed in this study was appropriate to explain AS HRQOL with 57.6% increase in explanation and adequate FI for confidence (except for AGI). Our results emphasize that depression should be a parameter since it significantly affects AS HRQOL. However, health awareness should be excluded because of poor evidence indicating its effect on AS HRQOL.

Pooling variables with different characteristics as a single latent variable is not justified. Aging can lower QOL and income while education can increase QOL. Personal characteristics should be considered in successive data because they have the same characteristics as other observed variables that measure a single latent variable. In this study, intensity of smoking was the most important key factor that could represent the characteristic of disease in “general characteristics”, making it acceptable as an observed variable. Similar to our study, a narrative Turkish AS HRQOL study (12) has shown no significant relation between ESR and HRQOL or between CRP and HRQOL.

In a study on relationship among pain, family support, and HRQOL, Lim and Moon have reported that family support shows relevant correlation with pain and strong relevant correlation with QOL (3). Therefore, these four areas of social support are relevant to pain.

Spouse support has indirect effect via sense of loss while medical support has no indirect or direct effect on women’s QOL after hysterectomy (37). In addition, social support has indirect effect on HRQOL via awareness and symptoms according to an obstructive sleep apnea study after substituting environmental with social support (38). Likewise, Oh and Lee have reported that environmental factors have significant indirect and direct effects on elderly QOL in patients with degenerative arthritis via symptom, function, and health awareness (39). Results of this study also showed no direct effect of environment support on HRQOL. However, it had significantly indirect effect on HRQOL through parameters of symptom, depression, and function.

Furthermore, there was a significant correlation (r = 0.619, *P* = 0.028) between fatigue and function, including significant correlation with physical role (r = −0.245, *P* = 0.044), vitality (r = −0.366, *P* = 0.002), general health (r = −0.390, *P* = 0.010), body pain (r = −0.436, *P* < 0.001), and mental health (r = −0.271, *P* = 0.025). Relevant co-relationship between quality of life and fatigue (co-relationship study between fatigue and other clinical factors) has been previous reported (16). This study also showed a significant correlation between fatigue and QOL (r = 0.37, *P* < 0.05).

Batmaz et al. have explained that QOL has a direct correlation with Quality of Sleep (QOS) (β = 0.265, *P* = 0.017) with 7% total change of effectiveness and a relevant co-relationship between subjective QOS and QOL (40). Although QOS is an influential factor indicating the need for objective measures and validation, it was excluded from our study as a bio-physiological factor. Nevertheless, QOS could be added as a parameter in future HRQOL studies of AS patients.

Our results showed that anxiety (*r* = 0.56, *P* = 0.0001) and depression (*r* = 0.60, *P* = 0.001) had correlation with QOL and function (anxiety: *r* = 0.40, *P* = 0.0001; depression: *r* = 0.39, *P* = 0.001). These results corroborated with an earlier report (28) showing a significant co-relationship between depression (*r* = 0.28, *P* < 0.001) and function. Depression (*r* = 0.48, *P* < 0.001) was correlated with HRQOL using the Korean Beck Depression Index-II (KBDI-II) method which included anxiety as a parameter.

Many previous studies have suggested a significant correlation between function and QOL. Therefore, function has sufficient theoretical basis as an influential factor for deciding ASHRQOL.

In a study on 75 AS patients, physical therapy has decreased disease activity from 38.7 to 26.7 points, functional limitation from 4.33 to 3.81 points, and pain level from 41.1 to 24.0 points (41). Another AS study has shown a 10.0-point decrease in disease activity and 7.3 points increase in health status a year later because of a 3-week rehab program (42). In this study, direct effect of physical activity on HRQOL through mediating parameters of symptoms, depression, and function was insignificant. Meanwhile, a significant indirect effect (β = −0.167, *P* = 0.007) on QOL was observed. Research on physical activity among AS patients and inter-study differences in either pre- or post-therapy programs is limited.

## Limitations

The following three study limitations have to be carefully considered for this study. First, population representation was a limitation because all participants were out-patients from a rheumatology clinic in Chonnam University Hospital. Second, there might be data bias because self-reporting questionnaire was used. Third, generalization was difficult because other factors beyond our control were not addressed in this study.

## Conclusion

As a result of the final revised model fit test, the model met recommended levels except for AGFI. The model explained 57.6% of ASQOL. Based on our study, the following recommendations are made to promote AS HRQOL. First, function and depression were identified as important factors affecting QOL. Therefore, peer therapy (e.g., AS anonymous or group-activities) that can manage depression in local settings should be considered. Consultation with psychiatrists should also be considered when needed. Second, our results confirmed that symptoms and physical activity were important factors for improving function and depression. Therefore, program should be developed to improve physical activity of AS patients. Additionally, environmental support, physical activity, and bio-physical factors were confirmed to be able to improve AS symptoms. Environmental support was more important than bio-physical and physical activity factors. Development of social welfare system and family support program could improve environmental support. Finally, interventional nursing programs that can manage these integral factors need to be developed to vigorously improve ASHRQOL.

## Ethical considerations

Ethical issues (Including plagiarism, informed consent, misconduct, data fabrication and/or falsification, double publication and/or submission, redundancy, etc.) have been completely observed by the authors.

## References

[1] Joel DT (2006). The spondyloarthritis. In HARRISON’S Principles of Internal Medicine (Yu D.H. ed.), MIP: Seoul, pp. 2175–2178.

[2] Gran JT Husby G (1984). Ankylosing Spondylitis: a comparative study of patients in an epidemiological survey, and those admitted to a department of rheumatology. J Rheumatol, 11(6): 788–793. 6520833

[3] Lim HJ Moon YI (1998). Pain, Family Support and Physical Activity in Patients with Ankylosing Spondylitis. Korea Society of Nursing Science, 28(2): 329–343.

[4] Braun J Sieper J Zink A (2012). The risks of smoking in patients with spondyloarthritides. Ann Rheum Dis, 71(6): 791–792. 22331652 10.1136/annrheumdis-2011-200954

[5] Chen CH Chen HA Lu CL (2013). Association of cigarette smoking with Chinese ankylosing spondylitis patients in Taiwan: a poor disease outcome in systemic inflammation, functional ability, and physical mobility. Clin Rheumatol, 32(5): 659–663. 23329350 10.1007/s10067-013-2165-y

[6] Chung HY Machado P van der Heijde D D'Agostino MA Dougados M (2012). Smokers in early axial spondyloarthritis have earlier disease onset, more disease activity, inflammation and damage, and poorer function and health-related quality of life: results from the DESIR cohort. Ann Rheum Dis, 71(6): 809–816. 21989541 10.1136/annrheumdis-2011-200180

[7] Ibn Yacoub Y Amine B Laatiris A Hajjaj-Hassouni N (2012). Gender and disease features in Moroccan patients with ankylosing spondylitis. Clin Rheumatol, 31(2): 293–297. 21796348 10.1007/s10067-011-1819-x

[8] Mattey DL Dawson SR Healey EL Packham JC (2011). Relationship between smoking and patient-reported measures of disease outcome in ankylosing spondylitis. J Rheumatol, 38(12): 2608–2615. 21965641 10.3899/jrheum.110641

[9] Martindale J Smith J Sutton CJ Grennan D Goodacre L Goodacre JA (2006). Disease and psychological status in ankylosing spondylitis. Rheumatology (Oxford) 45(10): 1288–1293. 16595514 10.1093/rheumatology/kel115

[10] Turan Y Duruöz MT Bal S Guvenc A Cerrahoglu L Gurgan A (2007). Assessment of fatigue in patients with ankylosing spondylitis. Rheumatol Int, 27(9): 847–852. 17252263 10.1007/s00296-007-0313-x

[11] Ozdemir O (2011). Quality of life in patients with ankylosing spondylitis: relationships with spinal mobility, disease activity and functional status. Rheumatol Int, 31(5): 605–610. 20049451 10.1007/s00296-009-1328-2

[12] Yılmaz O Tutoğlu A Garip Y Ozcan E Bodur H (2013). Health-related quality of life in Turkish patients with ankylosing spondylitis: impact of peripheral involvement on quality of life in terms of disease activity, functional status, severity of pain, and social and emotional functioning. Rheumatol Int, 33(5): 1159–1163. 22955799 10.1007/s00296-012-2510-5

[13] Oktayoglu P Em S Tahtasiz M (2013). Elevated serum levels of high mobility group box protein 1 (HMGB1) in patients with ankylosing spondylitis and its association with disease activity and quality of life. Rheumatol Int, 33(5): 1327–1331. 23143556 10.1007/s00296-012-2578-y

[14] Durcan L Wilson F Conway R Cunnane G O'Shea FD (2012). Increased body mass index in ankylosing spondylitis is associated with greater burden of symptoms and poor perceptions of the benefits of exercise. J Rheumatol, 39(12): 2310–2314. 23070993 10.3899/jrheum.120595

[15] Lim HJ Lim HS Lee MS (2005). Relationship between self-efficacy and exercise duration in patients with ankylosing spondylitis. Clin Rheumatol, 24(4): 442–443. 15338448 10.1007/s10067-004-0974-8

[16] Sandhu J Packham JC Healey EL Jordan KP Garratt AM Haywood KL (2011). Evaluation of a modified arthritis self-efficacy scale for an ankylosing spondylitis UK population. Clin Exp Rheumatol, 29(2): 223–230. 21504660

[17] Förger F Østensen M Schumacher A Villiger PM (2005). Impact of pregnancy on health related quality of life evaluated prospectively in pregnant women with rheumatic diseases by the SF-36 health survey. Ann Rheum Dis, 64(10): 1494–1499. 15778241 10.1136/ard.2004.033019 PMC1755222

[18] Oh JH (2013). Structural Equation Modeling on Quality of life in Older Adults with Osteoarthritis [dissertation]: Seoul Unversity Seoul. 10.4040/jkan.2014.44.1.75 24637288

[19] Bae ES (2014). A Prediction Model for Quality of Life in Adults Older with Parkinson’s Disease [dissertation]: Kyungsung University Busan.

[20] Wilson IB Cleary PD (1995). Linking Clinical variables with health-related quality of life: a conceptual model of patient outcomes. JAMA, 273(1): 59–65. 7996652

[21] Kiltz U van der Heijde D (2009). Health-related quality of life in patients with rheumatoid arthritis and in patients with ankylosing spondylitis. Clin Exp Rheumatol, 27(4 Suppl 55): S108–111. 19822055

[22] Marengo MF Schneeberger EE Citera G Cocco JA (2008). Work status among patients with ankylosing spondylitis in Argentina. J Clin Rheumatol, 14(5): 273–277. 18679137 10.1097/RHU.0b013e31817d1089

[23] Vesović-Potić V Mustur D Stanisavljević D Ille T Ille M (2009). Relationship between spinal mobility measures and quality of life in patients with ankylosing spondylitis. Rheumatol Int, 29(8): 879–884. 19172276 10.1007/s00296-008-0759-5

[24] Yu JP (2014). Structural equation modeling misunderstandings and prejudices of professors Jong Pil Yu. Data solution: Hanna Rae Seoul.

[25] Farren W Goodacre L Stigant M (2013). Fatigue in ankylosing spondylitis: causes, consequences and self-management. Musculoskeletal Care, 11(1): 39–50. 22825963 10.1002/msc.1029

[26] Sommerfleck FA Schneeberger EE Buschiazzo EE Maldonado Cocco JA Citera G (2012). A simplified version of Ankylosing Spondylitis Disease Activity Score(ASDAS) in patients with ankylosing spondylitis. Clin Rheumatol, 31(11): 1599–1603. 22895877 10.1007/s10067-012-2056-7

[27] Turan Y Duruöz MT Cerrahoglu L (2009). Relationship between enthesitis, clinical parameters and quality of life in spondyloarthritis. Joint Bone Spine, 76(6): 642–647. 19464222 10.1016/j.jbspin.2009.03.005

[28] Baysal O Durmuş B Ersoy Y (2011). Relationship between psychological status and disease activity and quality of life in ankylosing spondylitis. Rheumatol Int, 31(6): 795–800. 20221605 10.1007/s00296-010-1381-x

[29] Sung HM Kim JB Park YN Bai DS Lee SH Ahn HN (2008). A study on the reliability and the Validity of Korean Version of the Beck Depression Inventory-II(BDI-II). Journal Korean Soc Bid Therapy Psychiatry, 14(2): 201–212.

[30] Altan L Korkmaz N Dizdar M Yurtkuran M (2012). Effect of Pilates training on people with ankylosing spondylitis. Rheumatol Int, 32(7): 2093–2099. 21499876 10.1007/s00296-011-1932-9

[31] Karapolat H Eyigor S Zoghi M Akkoc Y Kirazli Y Keser G (2009). Are swimming or aerobic exercise better than conventional exercise in ankylosing spondylitis patients? A randomized controlled study. Eur J Phys Rehabil Med, 45(4): 449–457. 20032902

[32] Altan L Bingöl U Aslan M Yurtkuran M (2006). The effect of balneotherapy on patients with ankylosing spondylitis. Scand J Rheumatol, 35(4): 283–289. 16882592 10.1080/03009740500428806

[33] Shim JH (2009). Effectiveness of Exercise Therapy on Physical Function in Patients with Ankylosing Spodylitis: Systemic Review and Meta-Analysis. Korean Research Society of Physical Therapy, 16(3): 50–59.

[34] Lim MK (2002). Relationships between Social Support and Health among Low Income Groups in Urban Area [dissertation]: Seoul University Seoul.

[35] Park HJ Kim S Lee JE Jun JB Bae SC (2008). The reliability and validity of a Korean translation of the BASDAI in Korean patients with ankylosing spondylitis. Value Health, 11(1): S99–104. 18387074 10.1111/j.1524-4733.2008.00373.x

[36] Choi JH Kim TJ Shin K (2014). The Reliability and Validity of a Korean Translation of the ASAS Health Index and Environmental Factors in Korean Patients with Axial Spondyloarthritis. J Korean Med Sci, 29(3): 334–337. 24616580 10.3346/jkms.2014.29.3.334 PMC3945126

[37] Schwartz C Frohner R (2005). Contribution of demographic, medical, and social support variables in predicting the mental health dimension of quality of life among people with multiple sclerosis. Health Soc Work, 30(3): 203–212. 16190296 10.1093/hsw/30.3.203

[38] Choi SJ Kim KS (2013). Structural Equation Modeling On Health-related Quality of Life in Patients with Obstructive Sleep Apnea. J Korean Acad Nurs, 43(1): 81–90. 23563071 10.4040/jkan.2013.43.1.81

[39] Oh JH Yi MS, (2014). [Structural Equation Modeling on Quality of Life in Older Adults with Osteoarthritis]. J Korean Acad Nurs, 44(1): 75–85. 24637288 10.4040/jkan.2014.44.1.75

[40] Batmaz I Sariyildiz MA Dilek B Bez Y Karakoc M Cevik R (2013). Sleep quality and associated factors in ankylosing spondylitis: relationship with disease parameters, psychological status and quality of life. Rheumatol Int, 33: 1039–1045. 22940709 10.1007/s00296-012-2513-2

[41] Gyurcsik ZN András A Bodnár N Szekanecz Z Szántó S (2012). Improvement in pain intensity, spine stiffness, and mobility during a controlled individualized physiotherapy program in ankylosing spondylitis. Rheumatol Int, 32(12): 3931–3936. 22198694 10.1007/s00296-011-2325-9

[42] Kjeken I Bø I Rønningen A (2013). A three-week multidisciplinary in-patient rehabilitation programme had positive long-term effects in patients with ankylosing spondylitis: randomized controlled trial. J Rehabil Med, 45(3): 260–267. 23138412 10.2340/16501977-1078

